# Experimental and modeling investigation of mass transfer during combined infrared‐vacuum drying of Hayward kiwifruits

**DOI:** 10.1002/fsn3.435

**Published:** 2016-10-25

**Authors:** Emad Aidani, Mohammadhossein Hadadkhodaparast, Mahdi Kashaninejad

**Affiliations:** ^1^ Department of Food Science and TechnologyFerdowsi University of MashhadMashhadIran; ^2^ Faculty of Food ScienceGorgan University of Agricultural Sciences and Natural ResourcesGorganIran

**Keywords:** effective moisture diffusivity, image processing, infrared‐vacuum dryer, kiwifruit

## Abstract

In this work, we tried to evaluate mass transfer during a combined infrared‐vacuum drying of kiwifruits. Infrared radiation power (200–300 W) and system pressure (5–15 kPa), as drying parameters, are evaluated on drying characteristics of kiwifruits. Both the infrared lamp power and vacuum pressure affected the drying time of kiwifruit slices. Nine different mathematical models were evaluated for moisture ratios using nonlinear regression analysis. The results of regression analysis indicated that the quadratic model is the best to describe the drying behavior with the lowest *SE* values and highest *R* value. Also, an increase in the power led to increase in the effective moisture diffusivity between 1.04 and 2.29 × 10^−9^ m^2^/s. A negative effect was observed on the Δ*E* with increasing in infrared power and with rising in infrared radiation power it was increased. Chroma values decreased during drying.

## 1 Introduction

Kiwifruit (*Actinidia deliciosa*) or Chinese gooseberry is a fruit with a high level of vitamin C and phytonutrients including lutein, carotenoids, phenolics, chlorophyll, and flavonoids. Furthermore, shelf‐life of kiwifruit is very short and using a preservation methods is really necessary to extend its shelf‐life (Cassano, Figoli, Tagarelli, Sindona, & Drioli, 2006). Drying is an appropriate food preservation process (Shahraki, Jafari, Mashkour, & Emaeilzadeh, 2014). This process can increase their storage/shelf‐life and considered as a pretreatment for other processing such as frying (Aghilinategh, Rafiee, Hosseinpour, Omid, & Mohtasebi, 2015; Hashemi Shahraki, Ziaiifar, Kashaninejad, & Ghorbani, 2014; Naderinezhad, Etesami, Poormalek Najafabady, & Ghasemi Falavarjani, 2016).

Maskan (2001a) compared the hot air, microwave, and combined hot air‐microwave drying for kiwifruits samples with respect to rehydration characteristics and shrinkage. Chen, Pirini, and Ozilgen (2001) studied the simulation of making fruit leather. They established the drying kinetics parameters using obtained experimental data during pulped kiwifruit drying.

A suitable method to decrease the drying time is heating by infrared radiation. This infrared heating is appropriate for thin layers drying of samples with a large surface. In food processing, the infrared drying is conducted in radiator construction (Doymaz, 2014; Khir et al., 2014). The performance of these radiators is about 85% and the wavelength of emitted radiation is miniaturized (Nowak & Lewicki, 2004; Sandu, 1986). Transmitting of infrared through water leads to absorb the long wavelength (Sakai & Hanzawa, 1994). Infrared radiation is applied for cooking and heating cereal grains, vegetables, soybeans, seaweed, cocoa beans and nuts, processed meat (Nowak & Lewicki, 2004; Ratti & Mujumdar, 1995). Measurement of water content in food can be calculated using infrared drying (Nowak & Lewicki, 2004).

During vacuum drying of food the contact between the oxygen and sample is limited and it can be counted as a valuable advantage. Because of low pressure, the higher performance drying is expected even at low temperature (Ghaboos, Ardabili, Kashaninejad, Asadi, & Aalami, 2016; Nawirska, Figiel, Kucharska, Sokół‐Łętowska, & Biesiada, 2009). The combined infrared‐vacuum drying benefits both infrared heating and vacuum condition. Recently, infrared‐vacuum drying was used to dry the wide range of food products with high quality. The high rate mass transfer and low temperature can improve the energy efficiency of process and product quality (Giri & Prasad, 2007).

In order to successful industrial design of combined infrared‐vacuum drying system, it is necessary to investigate the drying characteristics under various condition (McLoughlin, McMinn, & Magee, 2003).

Infrared‐vacuum method can produce a high‐quality product (Salehi, Kashaninejad, Asadi, & Najafi, 2016). There for, the aim of our study was to investigate the combined infrared‐vacuum drying of kiwifruit slices with respect to moisture diffusivity, drying kinetics, and color changes.

## 2 Materials and Methods

### 2.1 Infrared‐vacuum drying

Kiwifruits (*Actinidia deliciosa*) were prepared from a local store. In order to decrease the respiration, the whole samples were stored at 4°C before using in experiments (Maskan, 2001b). The moisture content of kiwifruits was about 82% ±1.3 (wet basis). Before drying, all samples were peeled and cut into 0.5‐mm‐thick slices with a steel cutter.

**Figure 1:**
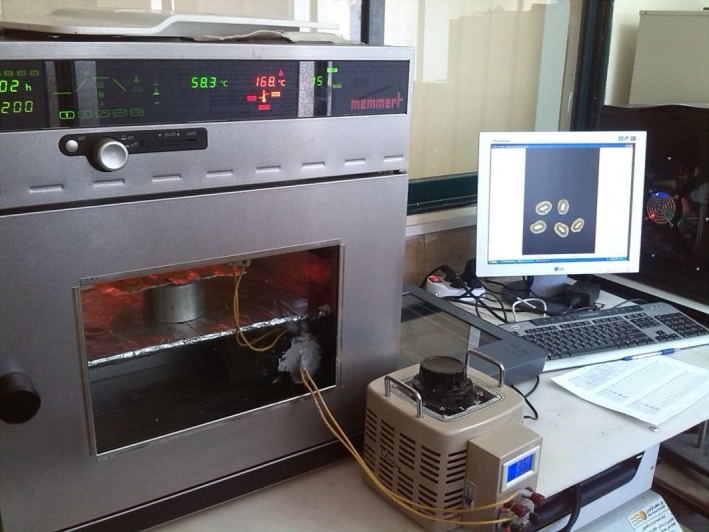
A schematic of the infrared‐vacuum dryer

A combined infrared (Philips, Germany) – vacuum (Memmert Universal, Germany) dryer was used to dry the kiwifruit slices (Figure 1). The drying was conducted in various power of infrared radiation (200, 250, and 300 W) and pressure (5, 10, and 15 kPa). The dried samples were stored in an airtight packet till the experiments (Ghaboos et al., 2016).

Weight loss was registered using a digital scale (LutronGM‐300p; Taiwan). The initial moisture content was determined based on the AOAC method (Helrich, 1990). All experiments were performed tree times and an the average was taken for data analysis (Ghaboos et al., 2016).

### 2.2 Kinetics of drying

The moisture content data were calculated by Equation (1):

(1) $$\text{MR}=\frac{M_{t}-M_{e}}{M_{0}-M_{e}}$$

where, MR: the dimensionless moisture ratio; *M*_*t*_: moisture content at any time *M*_0_: initial moisture content; *M*_e_: equilibrium moisture content.

The details of evaluated thin‐layer drying models, presented in Table 1, these models were fitted to obtained results for MR (Doymaz, 2014; Ghaboos et al., 2016). A nonlinear estimation package (Curve Expert, Version 1.34) was used to estimate the models coefficients. The correlation coefficient (*R*) and standard error (*SE*) were calculated to adjust the experimental results to the models A desirable fitness is achieved at low *SE* and high *R* values, (Doymaz, 2011).

**Table 1:** Applied mathematical models to kinetics modeling of kiwi drying

Model	Equation
Approximation of diffusion	MR = *a* exp(−*kt*) + (1 − *a*) exp(−*kat*)
Page	MR = exp(−*kt* ^*n*^)
Modified Page – II	MR = exp(−*c*(*t*/*l* ^2^)^*n*^)
Newton	MR = exp(−*kt*)
Midilli	MR = *a* exp(−*kt* ^n^) + *bt*
Logarithmic	MR = *a* exp(−*kt*) + *c*
Verma	MR = *a* exp(−*kt*)+(1 − *a*) exp(−*gt*)
Two term	MR = *a* exp(−*k*_0_ *t* ^*n*^) + *b* exp(−*k*_1_ *t*)
Quadratic	MR = *a* + *bx* + *cx* ^2^

MR, moisture ratio; *t*, time (min) and *n*,* k*,* b*,* l*,* g*,* c,* and *a* are coefficients of models.

### 2.3 Moisture diffusivity calculation

In drying, the diffusion is suggested as the main mechanism for the moisture transport to the surface (Doymaz, 2011). For food drying process, Fick's second law of diffusion has been widely introduced to describe a falling rate stage (Sacilik, 2007). This model is presented for slab geometry as Equation (2) (Ghaboos et al., 2016):

(2) $$\text{MR}=\frac{8}{\pi^{2}}\sum_{n=0}^{\infty }\frac{1}{{\left(2n+1\right)}^{2}}\exp\left(-{\left(2n+1\right)}^{2}\frac{\pi^{2}D_{\mathrm{eff}}t}{4L^{2}}\right)$$

where, MR: moisture ratio; *t*: drying time (*s*); *D*_eff_: effective diffusivity (m^2^/s); *L*: half slab thickness of slices (m). When the drying periods is too long, Equation (2) can be abbreviated to Equation (3) (Ghaboos et al., 2016).

(3) $$\text{MR}=\frac{8}{\pi^{2}}\exp\left[\frac{-\pi^{2}D_{\mathrm{eff}}t}{4L^{2}}\right]$$

The effective diffusivity can be obtained by Equation (3). It is typically calculated using plotting lnMR versus time (as given in Equation (3)) (Ghaboos et al., 2016). The slop of a straight line (*K*) in plot of lnMR versus time can obtained using Equation (3):

(4) $$K=\frac{\pi^{2}D_{\mathrm{eff}}}{4L^{2}}$$

### 2.4 Color measurement

An image processing system was used to determine the effect of drying condition on color indexes of dried kiwifruit, Sample images were captured with a scanner (Canon CanoScan LiDE 120; Japan). The color space of images was in RGB system and they were converted into L*a*b* system. In the L*a*b* space, the color perception is more uniform (Mashkour, Shahraki, Mirzaee, & Garmakhany, 2014; Salehi & Kashaninejad, 2014; Salehi et al., 2016).

Hue angle (H) of the samples was calculated as follows (Salehi & Kashaninejad, 2014):

H = tan^−1^ (b*/a*) when a* > 0 and b* > 0

H = 180° + tan^−1^ (b*/a*) when a* < 0

H = 360° + tan^−1^ (b*/a*) when a* > 0 and b* < 0

The color changes (Δ*E*) and Chroma calculated using Equations (5) and (6), respectively (Salehi & Kashaninejad, 2014):

(5) $$\Delta E=\sqrt{{\left(\Delta L^{*}\right)}^{2}+{\left(\Delta a^{*}\right)}^{2}+{\left(\Delta b^{*}\right)}^{2}}$$

(6) $$C^{*}=\sqrt{{\left(a^{*}\right)}^{2}+{\left(b^{*}\right)}^{2}}$$

In this study, Image J software (Ver.1.41; USA) was used to perform the image analysis of dried kiwifruit (Salehi & Kashaninejad, 2014).

## 3 Results and Discussion

### 3.1 Effect of drying condition

The absorption of infrared radiation by water content is the most important parameter, which affects drying rate. In general, infrared radiation can be absorbed by materials in the thin surface layer of sample (Ghaboos et al., 2016; Nowak & Lewicki, 2004). During drying, the radiation properties of exposed material is affected by removal of the water content, so the absorptivity of the sample is decreased due to increasing in the reflection of the waves.

**Figure 2:**
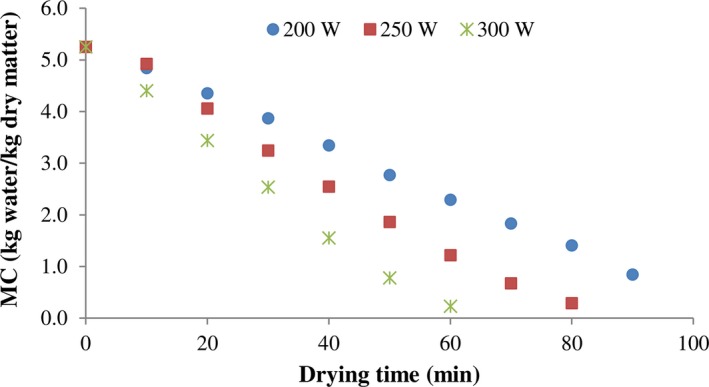
Variations of moisture content with drying time of kiwi slices at different infrared power (15 kPa)

**Figure 3:**
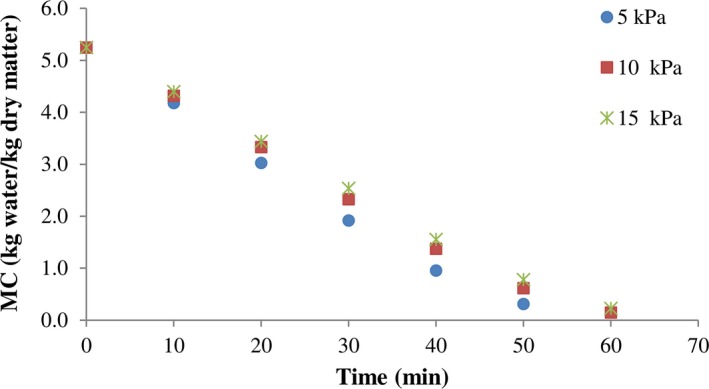
Variations of moisture content with drying time of kiwi slices at different system pressure (300 W)

Figures 2 and 3, present the changes in water content under studied infrared power and vacuum pressure, respectively. As can be seen, an increase in the power decreased the moisture content due to increasing temperature. In the fixed pressure (5 kPa), the drying periods of kiwifruit samples were 80, 60, and 47.5 min at 200, 250, and 300 W, respectively. Finally, the obtained results indicated that the power of infrared significantly affects the removal of moisture content.

In vacuum drying operation, drying is performed in low pressures. The reduction in temperature in the subatmospheric pressure leads to obtaining a higher quality compared to conventional air drying at atmospheric pressure (Ghaboos et al., 2016). With decreasing in the drying time from 92.5 to 80 min at a fixed infrared power, the vacuum pressure was decreased from 150 to 50 kPa (200 W). It seems that drying of thin layers had a higher efficiency at far‐infrared (25–100 μm) compared to near‐infrared radiation (NIR, 0.75–3.00 μm) for thicker samples (Salehi et al., 2016).

### 3.2 Drying curves fitting

**Figure 4:**
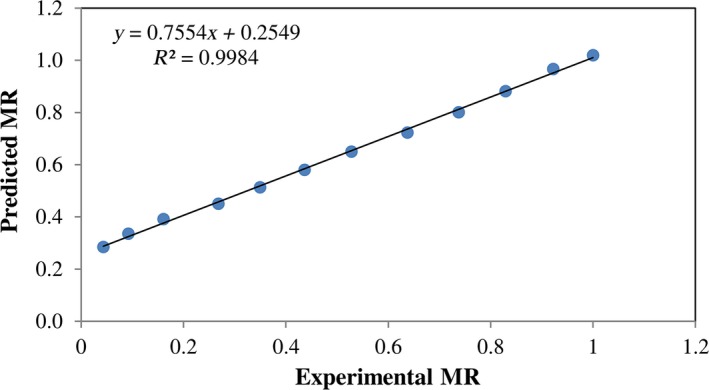
Comparison of experimental and predicted moisture ratio (MR) at 200 W and 15 kPa

The experimental data were fitted with the mathematical models (Table 1) and the quadratic model was the best model to describe the drying rate because it had the lowest *SE* and the highest *R* values. Statistical data obtained for this model and estimated parameters are presented in Table 2. The results indicated that for all models, the *R* values were higher than .997, stating a good correlation. Figure 4 shows the very good correlation between experimental and the predicted results using the quadratic model for dried kiwifruit slices at 200 W and 15 kPa.

**Table 2:** Curve‐fitting coefficients of the quadratic model

Power (W)	Pressure (kPa)	*a*	*b*	*C*	*R*	*SE*
200	5	1.015	−0.013	3.242 × 10^−05^	.999	0.011
200	10	1.022	−0.011	2.622 × 10^−05^	.998	0.019
200	15	1.019	−0.010	1.061 × 10^−05^	.999	0.014
250	5	1.011	−0.019	7.107 × 10^−05^	.999	0.015
250	10	1.014	−0.017	6.256 × 10^−05^	.999	0.016
250	15	1.040	−0.014	2.935 × 10^−05^	.997	0.028
300	5	1.011	−0.024	9.803 × 10^−05^	.999	0.018
300	10	1.016	−0.021	8.047 × 10^−05^	.998	0.021
300	15	1.014	−0.019	5.130 × 10^−05^	.998	0.019

### 3.3 Moisture diffusivity

**Figure 5:**
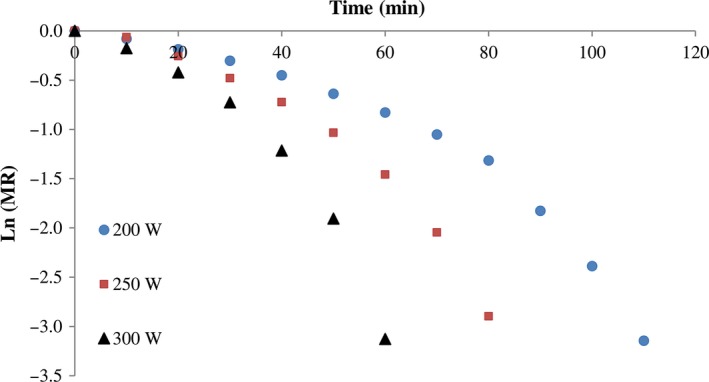
Effect of infrared power on the ln(MR) during drying of kiwi slices at 15 kPa system pressure. MR, moisture ratio

**Figure 6:**
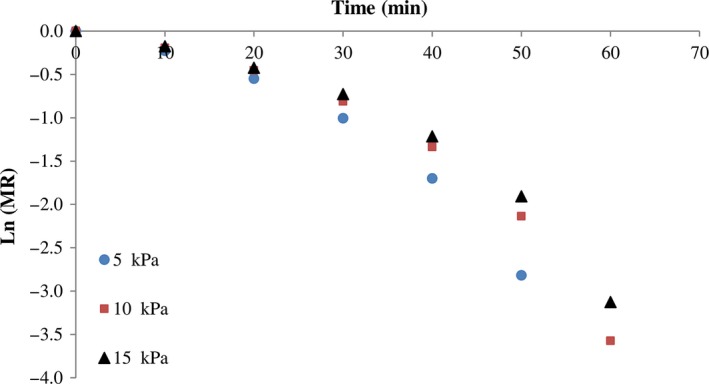
Effect of system pressure on the ln(MR) during drying of kiwi slices at 200 W power. MR, moisture ratio

The parameter of effective diffusivities was obtained using plotting lnMR versus time. The changes in lnMR under various infrared radiation power, vacuum pressure, and thickness are presented in Figures 5 and 6, respectively. The *D*_eff_ values for food samples are in range 10^−11^ to 10^−9^ m^2^/s (Doymaz & Göl, 2011). The values of *D*_eff_ at different condition drying of kiwifruit slice obtained by Equation (4) and predicted results are indicated in Table 3. The effective diffusivity of kiwifruit samples were obtained from 1.04 to 2.29 × 10^−9^ m^2^/s. This parameter increased with an increase in infrared radiation power due to high mass transfer at high temperatures (Ghaboos et al., 2016). Similar results were reported for hull‐less seed pumpkin (0.85 to 1.75 × 10^−10^ m^2^/s at 40–60°C) (Ghaboos et al., 2016; Sacilik, 2007), carrot in the (0.46–3.45 × 10^−10^ m^2^/s at 60–90°C) (Zielinska & Markowski, 2007), kiwifruit (3.0 to 17.12 × 10^−10^ m^2^/s at 30–90°C) (Simal, Femenia, Garau, & Rosselló, 2005), red bell pepper (3.2 to 11.2 × 10^−9^ m^2^/s at 50–80°C) (Vega, Fito, Andrés, & Lemus, 2007), curd (2.52 to 13.0 × 10^−10^ m^2^/s at 45–50°C) (Shiby & Mishra, 2007), and okra (4.27 to 13.0 × 10^−10^ m^2^/s at 50–70°C) (Doymaz, 2005).

**Table 3:** Values of effective moisture diffusivity of kiwi slice obtained from drying experiments

Power (W)	Pressure (kPa)	Effective diffusivity (m^2^/s)	*R*
200	5	1.08 × 10^−09^	.967
200	10	1.17 × 10^−09^	.951
200	15	1.04 × 10^−09^	.941
250	5	1.75 × 10^−09^	.960
250	10	1.79 × 10^−09^	.947
250	15	1.42 × 10^−09^	.954
300	5	2.25 × 10^−09^	.959
300	10	2.29 × 10^−09^	.940
300	15	2.00 × 10^−09^	.945

### 3.4 Color measurement

Color is an important quality factor for food production (Shahraki, Mashkour, & Garmakhany, 2014). The fresh kiwifruit exhibited a yellow color, with L*, a*, and b* equal to 50.98, −10.61, and 33.06, respectively. The obtained results for color measurement at various conditions indicated that infrared radiation power has a considerable effect on the color of kiwifruit slices (Table 4). With increasing in power of infrared from 200 to 300 W, Δ*E* was increased from 13.81 to 17.29, respectively. With respect to presented results in Table 4, the L* values changed from 38.65 to 49.73 at various drying condition. During drying process, the chroma values showed a decrease and a similar trend to the *b*‐values. The obtained value for chroma shows the saturation degree of color and is corresponding to the color strength (Maskan, 2001b). The variation in Hue angle values was not considerable compared to drying processes. Ghaboos et al. (2016) found that high temperature is responsible for increasing Δ*E* values during drying of mint leaves.

**Table 4:** Comparison between different drying methods on color change in kiwi slices

Power (W)	Pressure (kPa)	a*	b*	L*	Δ*E*	C*	Hue value (°)
200	5	−0.84 ± 3.85	34.49 ± 9.61	49.73 ± 3.64	9.95	34.50	91.39
200	10	−1.14 ± 3.72	34.32 ± 10.41	47.56 ± 2.00	10.15	34.34	91.89
200	15	0.24 ± 4.35	35.00 ± 10.82	42.66 ± 2.45	13.81	35.00	89.60
250	5	−0.40 ± 3.76	33.75 ± 12.05	41.67 ± 4.18	13.84	33.75	90.67
250	10	−0.47 ± 3.78	33.33 ± 12.21	41.03 ± 4.62	14.21	33.33	90.81
250	15	−1.18 ± 4.28	30.51 ± 10.21	40.51 ± 3.16	14.31	30.53	92.22
300	5	−0.79 ± 3.84	30.84 ± 12.39	40.39 ± 3.65	14.61	30.85	91.47
300	10	1.03 ± 4.19	31.20 ± 12.54	39.32 ± 4.30	16.58	31.22	88.11
300	15	0.30 ± 4.17	27.76 ± 13.23	38.65 ± 3.95	17.29	27.77	89.39

## 4 Conclusions

Kiwifruit samples were dried using a combined infrared‐vacuum dryer. The dryer was Equipped with near‐infrared (NIR) heaters. The drying times of kiwifruit were 80, 60, and 47.5 min at 200, 250, and 300 W, respectively. It was reduced when the system pressure was decreased. The drying kinetics were described by quadratic model with the latter providing the best representation of the experimental data. It was observed that the obtained effective moisture diffusivity values for kiwifruit samples were from 1.04 and 2.29 × 10^−9^ m^2^/s. This study verified that the color of kiwifruit was affected by the parameters of drying process. An increase in infrared radiation power from 200 to 300 W leads to increasing in Δ*E* from 13.81 to 17.29, respectively. The values for Hue angle changes were not considerable in comparison with drying processes.

## Conflict of Interest

None declared.
